# Supplementary material for: Postnatal cytomegalovirus infection and its effect on hearing and neurodevelopmental outcomes among infants aged 3–10 months: A cohort study in Eastern Uganda
Source: PLoS One. 2025 Feb 6;20(2):e0318655. doi: 10.1371/journal.pone.0318655 (PMC11801545; doi:10.1371/journal.pone.0318655)
Supplement: S1 Appendix — (PDF) [file pone.0318655.s001.pdf]

Supplement 1

CMV LABORATORY PROTOCOL

Molecular Diagnostics

A. Extraction Procedure

- Add enzymatic DNA/RNA extraction buffer 10x at 1:10 v/v buffer to sample (e.g 20 µL of buffer to 180 µL of sample).
- Mix completely by vortexing for 15 seconds.
- Incubate the mixture at room temperature for minimally 15 minutes.
- Incubate the sample at 98°C for 5 minutes.
- Centrifuge the lysate for 30 seconds to pellet cellular debris at 7000 rpm for 1 minute and utilize supernatant for PCR reaction.
- Store at -20°C temporarily, and -80°C for long term.

B. TaqMan PCR Assay Preparation

- Before use, suitable amounts of all TaqMan PCR components should be completely thawed at room temperature, mixed by gentle vortexing or by pipetting, and centrifuged briefly.
- For every TaqMan PCR run, one reaction containing CMV Positive Control and one reaction as no template control must be included for proper interpretation of results.
- To avoid any contamination while preparing the TaqMan PCR assay, follow the order outlined in Tables 1, 2 and 3 below to prepare the Negative Control, Detection Assay and Positive Control:
  1. Prepare the PCR Negative Control (Table 1)
  2. Prepare the PCR CMV Assay (Table 2)
  3. Prepare the PCR Positive Control (Table 3)
- To further avoid contamination, add the components to the PCR tubes in the order shown in the tables below i.e: 1) MDx TaqMan 2X PCR Master Mix; 2) Primer & Probe Mix; 3) TERT Taqman Assay; 4) Nuclease-free water; and 5) the Sample DNA or Positive Control).

C. Prepare the PCR Negative Control (Table 1)

Table 1. TaqMan PCR Negative Control Preparation

**PCR Components**
Nuclease-Free Water
MDx TaqMan 2x PCR Master Mix
CMV Primer & Probe Mix
Total Volume

D. Prepare the PCR CMV Assay (Table 2)

Table 2. TaqMan PCR CMV Assay Preparation: 1Rx

**PCR Components**	**Target detection (with MDx TaqMan 2x PCR Master Mix)**
MDx TaqMan 2x PCR Master Mix	10 µL
CMV Primer & Probe Mix	2 µL

TERT TaqMan Assay	2 µL
Sample DNA*	3 µL
Nuclease-Free Water	3 µL
**Total Volume**	**20 µL**

** The recommended amount of sample DNA to be used is 3 µL. However, a volume between 1 and 5 µL of sample DNA may be used as template. Adjust the final volume of the PCR reaction to 20 µL using the Nuclease-Free water provided.*

E. Prepare the PCR Positive Control (Table 3)

Table 3. TaqMan PCR Positive Control Preparation

**PCR Components**	**Target detection (with MDx TaqMan 2x PCR Master Mix)**
MDx TaqMan 2x PCR Master Mix	10 µL
CMV Primer & Probe Mix	2 µL
CMV Positive Control (PosC)	8 µL
Total Volume	20 µL

Reagent Preparation and Volume Required for 1 Run – 17 Reactions

TaqMan PCR CMV Assay Preparation : 17Rx

**PCR Components**	**Target detection (with MDx TaqMan 2x PCR Master Mix)**
MDx TaqMan 2x PCR Master Mix	170 µL
CMV Primer & Probe Mix	34 µL
TERT TaqMan Assay	34µL
Nuclease-Free Water	51 µL

Laboratory results, Laboratory Assays, units and reference ranges

A. CMV TaqMan PCR Assay Programming

1. Program the thermocycler according to the program shown in Table 4 below.
2. Run one step PCR.

Table 4. CMV TaqMan PCR Program

One Step PCR Cycle	Step	Temperature	Duration
Cycle 1	**Step 1**	**50ºC**	**30 min**
Cycle 2	**Step 1**	**95ºC**	**3 min**
Cycle 3 (40x)	**Step 1**	**95ºC**	**15 sec**
	**Step 2**	**60ºC**	**30 sec**

B. CMV TaqMan PCR Assay Interpretation

Table 5. Interpretation of Assay Results

FAM (Target detection)	HEX (PCR validation)	Result
+	+	**Positive**
-	+	**Negative**
-	-	**PCR inhibited**

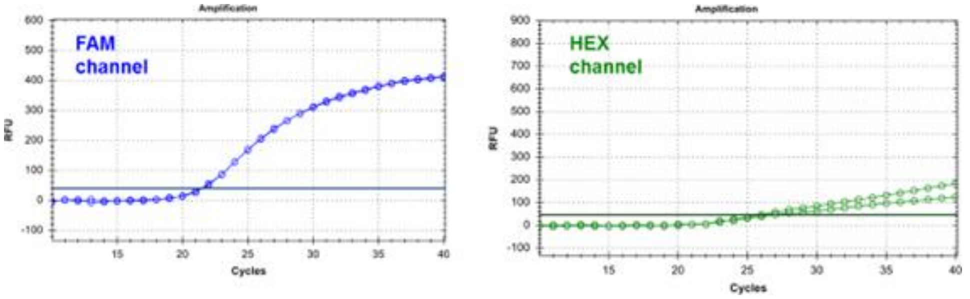

Figure 1. *Example of TaqMan PCR Positive result. Both PCR signals above the baseline fromFAM and HEX channel indicate the successful PCR.*

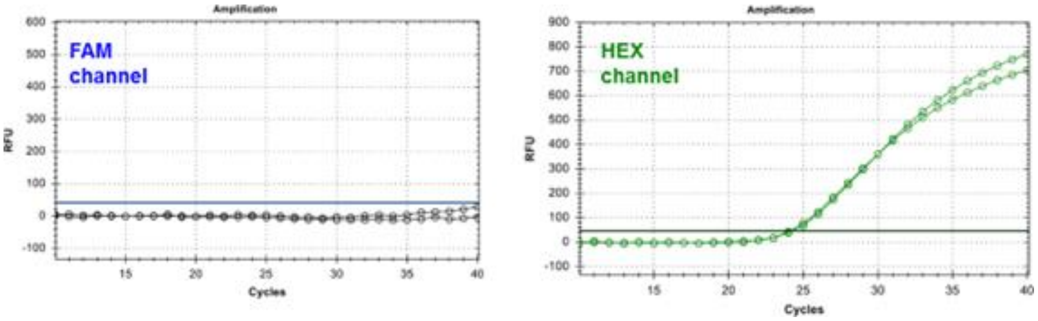

Figure 2. *Example of TaqMan PCR Negative result. No target DNA was detected in FAMchannel but amplification signal from HEX indicates the successful PCR.*

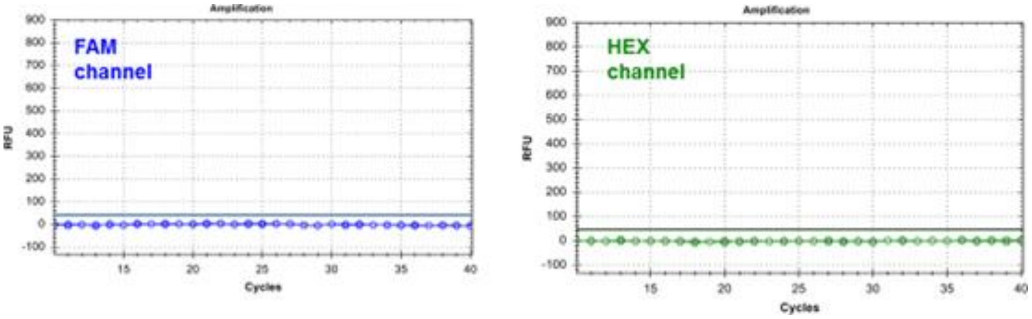

Figure 3. *Example of TaqMan PCR inhibition result. No signal from both FAM and HEX channel was detected. It is suggested to repeat the sample preparation using recommended kitfor DNA purification.*

CMV TaqMan PCR Assay Specificity

The specificity of Norgen's Cytomegalovirus (CMV) TaqMan PCR Kit is first and foremost, ensured by the selection of the CMV-specific primers, as well as the selection of stringent

reaction conditions. The CMV primers were checked for possible homologies in GenBank published sequences by sequence comparison analyses.

DETAILS OF MACHINES IN USE

BRAND: CHAI OPEN QPCR
