# Supplementary material for: Postnatal cytomegalovirus infection and its effect on hearing and neurodevelopmental outcomes among infants aged 3–10 months: A cohort study in Eastern Uganda
Source: PLoS One. 2025 Feb 6;20(2):e0318655. doi: 10.1371/journal.pone.0318655 (PMC11801545; doi:10.1371/journal.pone.0318655)
Supplement: S3 Appendix — (PDF) [file pone.0318655.s003.pdf]

Supplementary Tables (Exploratory)

a. Developmental Milestones by Age Group: Individual Gross Normative Domain Scores from the Malawi Development Assessment Tool (MDAT)

Age (Months, days)	N	Gross Motor (Av/Mean)	Fine Motor (Av/Mean)	Language (Av/Mean)	Social (Av/Mean)
**3 months (<= 121)**	128	5.58	3.86	4.20	5.20
**4 months (122 - 152)**	78	8.29	6.55	4.96	6.08
**5 months (153 - 182)**	64	9.73	8.52	5.72	7.17
**6 months (183 - 213)**	75	10.55	9.01	6.07	7.69
**7 months (214 - 243)**	31	11.42	9.84	6.45	8.42
**8 months (244 - 273)**	27	12.33	10.37	6.70	9.70
**9 months (274 - 304)**	15	12.53	10.06	6.20	10.53
**10 months (>= 305)**	6	12.67	11.17	7.33	10.33
**Total/Overall (3 -10 months)**	424	8.78	7.14	5.33	6.89

b. MDAT DAZ Outcome by individual domain

Outcome	Gross motor	Fine motor	Language	Social	Full model (overall)
**Fail**	0	4.68	0.94	0.47	2.11
**Pass**	100	95.32	99.06	99.53	97.89

c. MDAT DAZ Outcome by individual domain and CMV status

i. CMV Positive

Outcome	Gross motor	Fine motor	Language	Social	Full model (overall)
**Fail**	0	5.74	1.44	0.48	2.87
**Pass**	100	94.26	98.56	99.52	97.13

ii. CMV Negative

Outcome	Gross motor	Fine motor	Language	Social	Full model (overall)
**Fail**	0	3.67	0.46	0.46	1.38
**Pass**	100	96.33	99.54	99.54	98.62

d. Distribution of MDAT Z Scores by Postnatal CMV Status in Infants

	Total			Negative			Positive		
**Z score**	**N**	**Mean**	**SD**	**N**	**Mean**	**SD**	**N**	**Mean**	**SD**
**-3 to <-2**	9	-2.26	0.17 (-2.30, -2.16)	3	-2.16	0.07 (-2.2, -2.08)	6	-2.30	0.19 (-2.45, -2.15)
**-2 to <-1**	55	-1.31	0.24 (-1.44, -1.27)	34	-1.32	0.23 (-1.39, -1.23)	21	-1.29	0.27 (-1.36, -1.10)
**-1 to <0**	167	-0.39	0.26 (-0.57, - 0.15)	81	-0.38	0.25 (-0.43, -0.32)	86	-0.40	0.28 (-0.60, -0.14)
**0 to <1**	163	0.37	0.27 (0.34, 0.53)	83	0.34	0.25 (0.29, 0.40)	80	0.40	0.29 (0.36, 0.62)
**1 to <2**	30	1.37	0.27 (0.13, 0.53)	17	1.36	0.22 (1.25, 1.46)	13	1.38	0.27 (1.19, 1.63)
**-3 to <2**	424	-0.13	0.79 (-0.56, 0.36)	218	-0.14	0.79 (-0.24, -0.03)	206	-0.12	0.79 (-0.55, 0.37)

e. Neurological Assessment: Age-Stratified Hammersmith Infant Neurological Examination (HINE) Scores (By months)

Age	N	HINE Mean Score (95% CI)	HINE Above age specific threshold (%)
3 months (<=121)	128	64.12 (62.88, 65.37)	42.9
4 months (122 - 152)	78	65.33 (63.90, 66.76)	28.2
5 months (153 - 182)	64	69.75 (68.46, 71.02)	60.9
6 months (183 - 213)	75	72.18 (71.38, 73.00)	81.3
7 months (214 - 243)	31	74.29 (73.23, 75.35)	93.6
8 months (244 - 273)	27	74.15 (72.64, 75.65)	89.3
9 months (274 - 304)	15	73.53 (70.43, 76.64)	73.0
10 months (>=305)	6	74.67 (73.09, 76.25)	100.0
Overall (3 – 10 months)	424	68.49 (67.82, 69.14)	58.5

f. Neurological Assessment: Age-Stratified Hammersmith Infant Neurological Examination (HINE) Scores (By months and by CMV status)

i. CMV Positive

Age	N	HINE Mean Score (95% CI)	HINE Above age-specific threshold (%)
3 months (<=121)	56	64.58 (63.12, 66.04)	41.1
4 months (122 - 152)	40	65.80 (64.26, 67.33)	27.5
5 months (153 - 182)	35	69.40 (67.57, 71.22)	62.9
6 months (183 - 213)	35	72.40 (71.23, 73.56)	80.0
7 months (214 - 243)	15	74.80 (73.55, 76.05)	93.3
8 months (244 - 273)	14	74.50 (72.12, 76.88)	92.9
9 months (274 - 304)	8	72.25 (66.73, 77.77)	62.5

10 months (>=305)	3	74.67 (72.95, 76.38)	100.0
Overall (3 – 10 months)	206	68.83 (67.99, 69.66)	57.8

ii. CMV Negative

Age	N	HINE Mean Score (95% CI)	HINE Above age-specific threshold (%)
3 months (<=121)	72	63.70 (61.86, 65.66)	44.4
4 months (122 - 152)	38	64.84 (62.37, 67.31)	28.9
5 months (153 - 182)	29	70.17 (68.39, 71.95)	58.6
6 months (183 - 213)	40	72.00 (70.84, 73.16)	82.5
7 months (214 - 243)	16	73.81 (72.12, 75.50)	93.8
8 months (244 - 273)	13	73.76 (71.91, 75.63)	92.3
9 months (274 - 304)	7	75.00 (72.91, 77.10)	85.7
10 months (>=305)	3	74.67 (71.57, 77.76)	100.0
Overall (3 – 10 months)	218	68.16 (67.15, 69.16)	59.2

***Pass scores for infants aged 2 to 3 months, 4 to 9 months, and 9 to 24 months are defined as 67 or above, 70 or above, and 73 or above, respectively**

g. HINE AND MDAT – COMPARATIVE MATRIX

Age	N	MDAT Greater -2 (%)	HINE Above age specific threshold (%)
3 months (<=121)	128	100.0	42.9
4 months (122 - 152)	78	100.0	28.2
5 months (153 - 182)	64	98.4	60.9
6 months (183 - 213)	75	94.6	81.3
7 months (214 - 243)	31	96.7	93.6
8 months (244 - 273)	27	96.4	89.3
9 months (274 - 304)	15	87.5	73.0
10 months (>=305)	6	100.0	100.0
Overall (3 – 10 months)	424	97.8	58.5
