# Supplementary material for: Postnatal cytomegalovirus infection and its effect on hearing and neurodevelopmental outcomes among infants aged 3–10 months: A cohort study in Eastern Uganda
Source: PLoS One. 2025 Feb 6;20(2):e0318655. doi: 10.1371/journal.pone.0318655 (PMC11801545; doi:10.1371/journal.pone.0318655)
Supplement: S4 Appendix — (PDF) [file pone.0318655.s004.pdf]

SUPPLEMENTARY FIGURES

i. Malawi Development Assessment Tool (MDAT): Full model DAZ and scaled scores distribution.

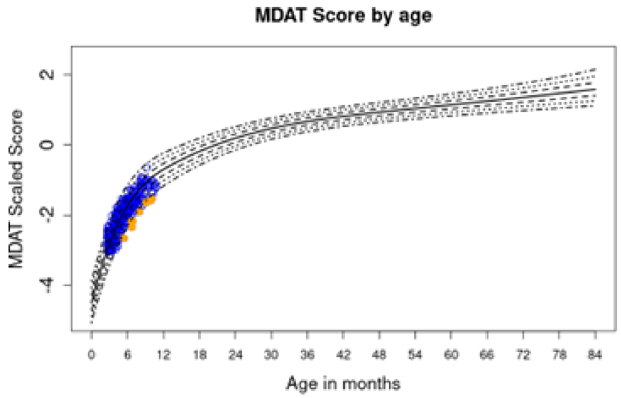

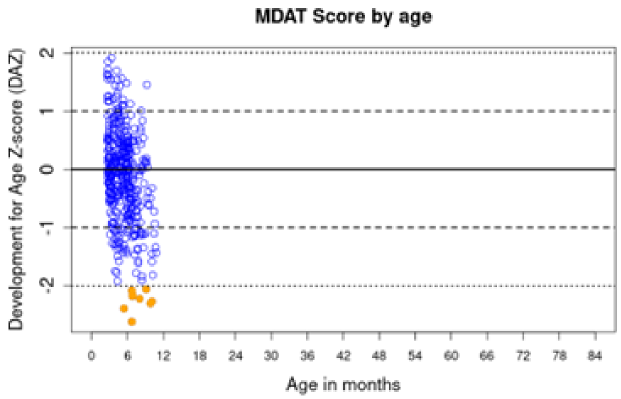

ii. Median Kernel density distribution (MDAT Overall)

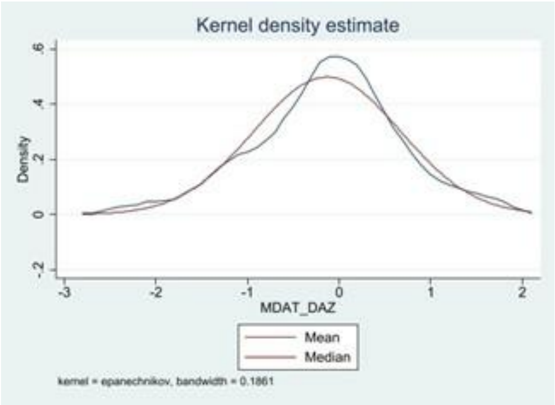

iii. Gross motor domain DAZ and scaled score distribution

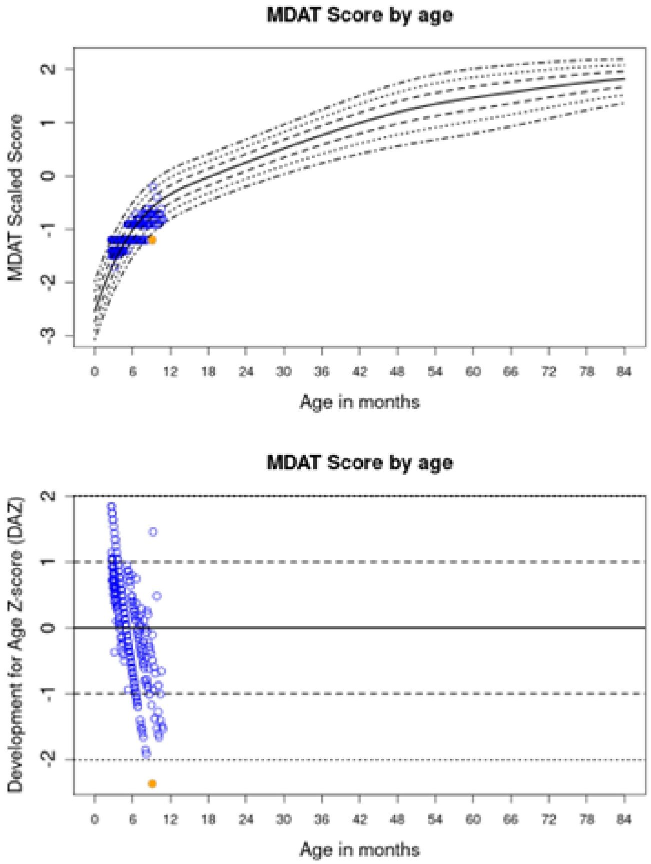

iv. Fine motor domain DAZ and scaled score distribution

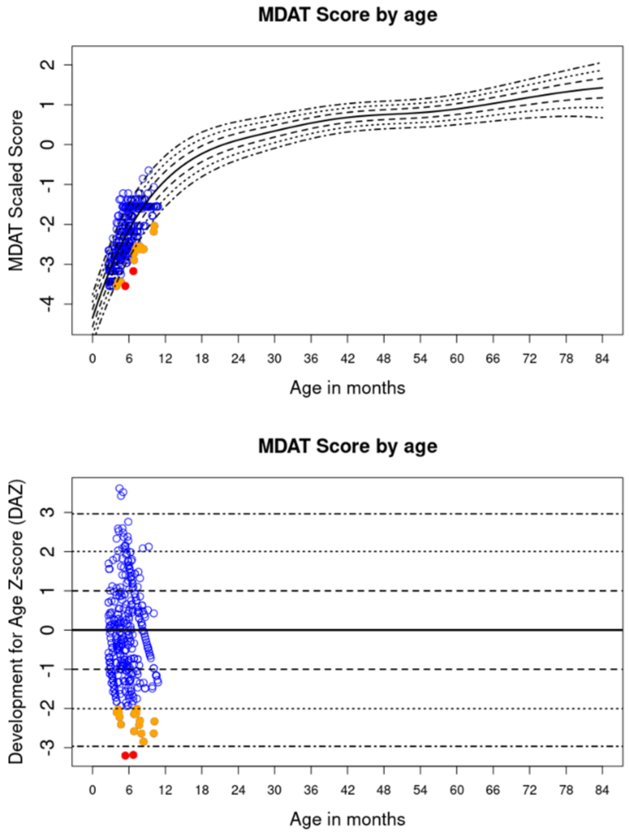

v. Language domain DAZ and scaled score distribution

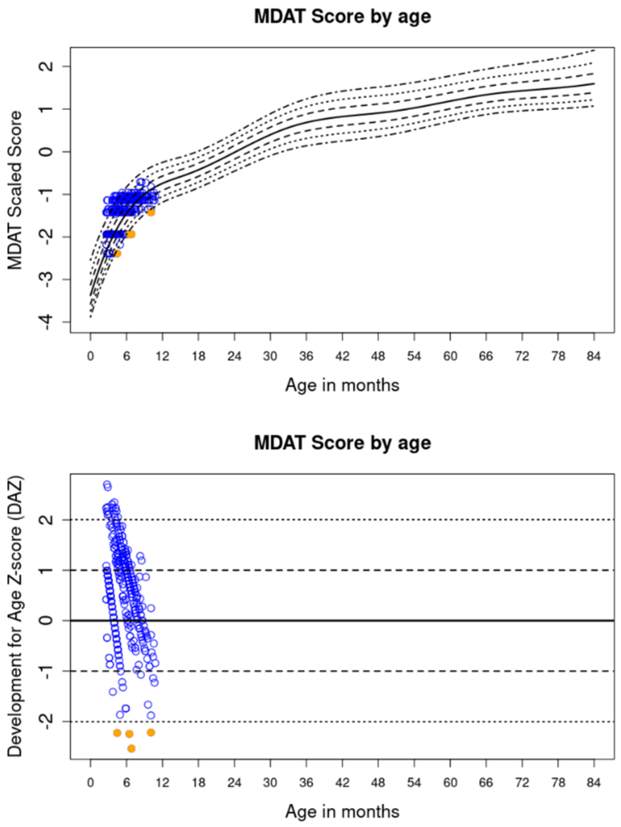

vi. Social domain DAZ and scaled score distribution

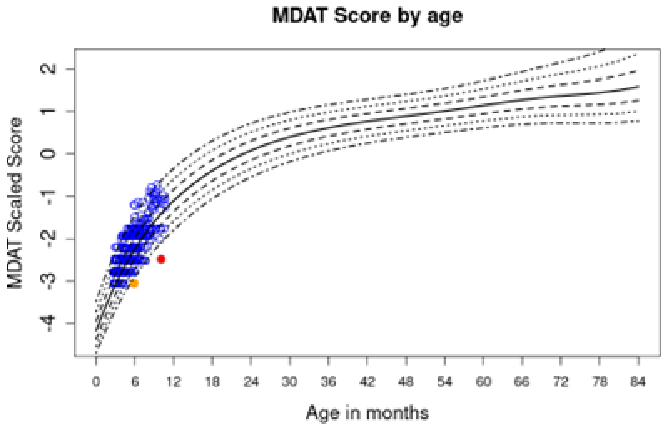

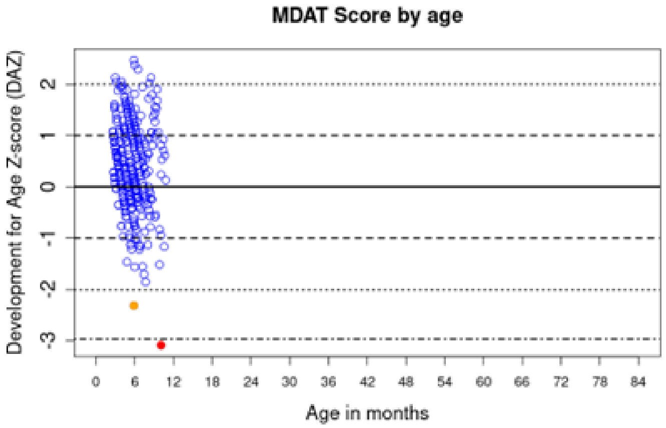

Histogram and boxplot MDAT

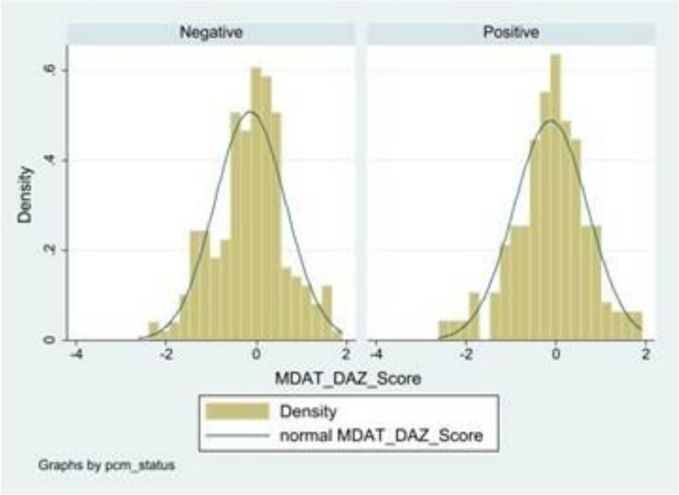

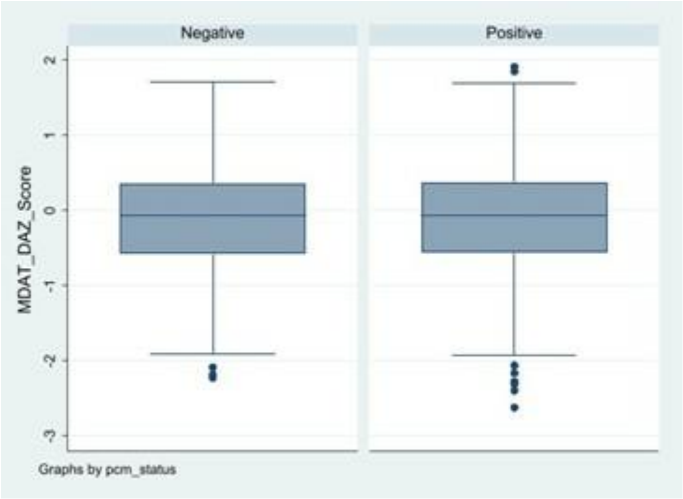

Histograms and boxplot HINE

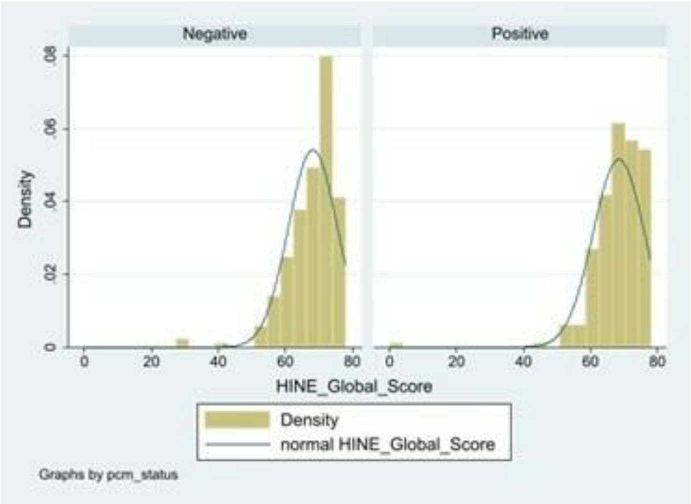

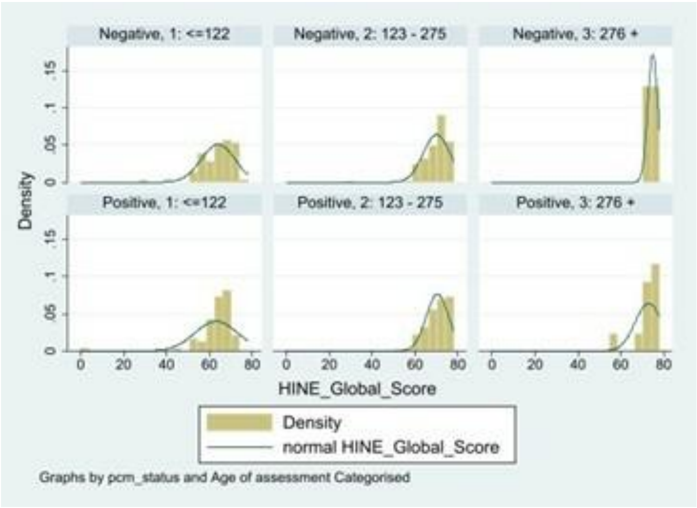

Box - plot HINE (Age- stratified)

Figure 4: Hearing Outcome

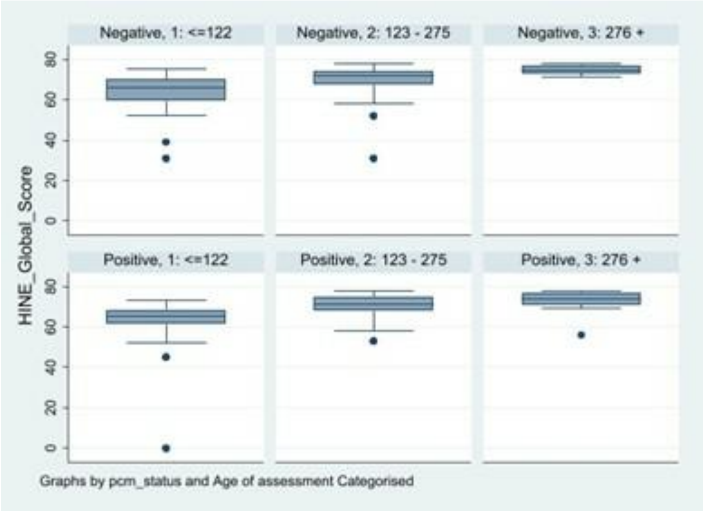
Negative, 1: <=122
Negative, 2: 123 - 275
Negative, 3: 276 +
Positive, 1: <=122
Positive, 2: 123 - 275
Positive, 3: 276 +
HINE_Global_Score
0
20
40
60
80
Graphs by pcm_status and Age of assessment Categorised

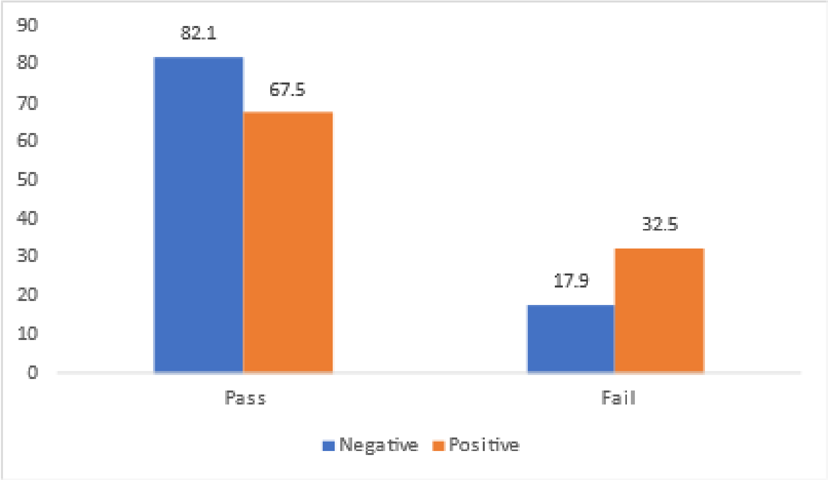
90
80
70
60
50
40
30
20
10
0
82.1
67.5
32.5
17.9
Pass
Fail
Negative
Positive
